# Runaway brain‐culture coevolution as a reason for larger brains: Exploring the “cultural drive” hypothesis by computer modeling

**DOI:** 10.1002/ece3.6350

**Published:** 2020-05-20

**Authors:** Alexander V. Markov, Mikhail A. Markov

**Affiliations:** ^1^ Faculty of Biology Moscow State University Moscow Russia; ^2^ Paleontological Institute of the Russian Academy of Sciences Moscow Russia

**Keywords:** brain expansion, cultural drive, human evolution, simulation, social learning

## Abstract

Scale and tempo of brain expansion in the course of human evolution implies that this process was driven by a positive feedback. The “cultural drive” hypothesis suggests a possible mechanism for the runaway brain‐culture coevolution wherein high‐fidelity social learning results in accumulation of cultural traditions which, in turn, promote selection for still more efficient social learning. Here we explore this evolutionary mechanism by means of computer modeling. Simulations confirm its plausibility in a social species in a socio‐ecological situation that makes the sporadic invention of new beneficial and cognitively demanding behaviors possible. The chances for the runaway brain‐culture coevolution increase when some of the culturally transmitted behaviors are individually beneficial while the others are group‐beneficial. In this case, “cultural drive” is possible under varying levels of between‐group competition and migration. Modeling implies that brain expansion can receive additional boost if the evolving mechanisms of social learning are costly in terms of brain expansion (e.g., rely on complex neuronal circuits) and tolerant to the complexity of information transferred, that is, make it possible to transfer complex skills and concepts easily. Human language presumably fits this description. Modeling also confirms that the runaway brain‐culture coevolution can be accelerated by additional positive feedback loops via population growth and life span extension, and that between‐group competition and cultural group selection can facilitate the propagation of group‐beneficial behaviors and remove maladaptive cultural traditions from the population's culture, which individual selection is unable to do.

## Graphical abstract

Extremely rapid brain expansion in the course of human evolution implies that this process was driven by a positive feedback. The “cultural drive” hypothesis suggests a possible mechanism for the runaway brain‐culture coevolution wherein high‐fidelity social learning results in accumulation of cultural traditions which, in turn, promote selection for still more efficient social learning. Here we explore this evolutionary mechanism by means of computer modeling and show that the conditions necessary for a powerful “cultural drive” are compatible with the current knowledge of the ecology and social organization of the Pleistocene Homo species.

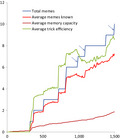

## 1 INTRODUCTION

One of the most intriguing features of hominin evolution is the rapid increase in brain volume, both absolute and relative, which was especially pronounced during the last two million years in the *Homo* clade. Within this time interval, brain volume increased threefold, from approximately 400–500 cm^3^ in the ancestral australopiths to 1,300–1,500 cm^3^ in the Late Pleistocene species such as Neanderthals and early modern humans (Holloway, 2015; Leigh, 2012; Neubauer & Hublin, 2012; Rightmire, 2004; Roth & Dicke, 2005; Schwartz, Holloway, Broadfield, Tattersall, & Yuan, 2004; Sherwood, Subiaul, & Zawidzki, 2008). This striking pattern of directional and accelerating evolution toward larger brains appears to be quantitatively unique among primates (Miller, Barton, & Nunn, 2019). Given that a large brain is very expensive metabolically and imposes other costs, for example, increased load on the cervical region of the spine and difficulties at giving birth to larger‐headed offspring (Gavrilets, 2015; Leonard & Robertson, 1992; Mink, Blumenschine, & Adams, 1981), its continued expansion during two million years of human evolution implies unusually strong and long‐lasting selection pressures in favor of larger‐brained individuals (or groups containing such individuals). This, in turn, almost inevitably implies a positive feedback loop in the evolution of the human brain: Its expansion must have been promoting further expansion (Crespi, 2004; Holloway, 1967; Miller et al., 2019).

Several kinds of hypothetical feedback mechanisms have been suggested in this context including sexual selection for intelligence accelerated by Fisherian runaway process (Miller, 2000), within‐group competition for social status which boosted the evolution of increasingly elaborate “Machiavellian intelligence” (Byrne & Whiten, 1988; Humphrey, 1976), and ever‐increasing between‐group competition in a highly social, ecologically dominant species which resulted in accelerated evolution of cognitive abilities needed for effective within‐group cooperation and group‐beneficial behaviors (Alexander, 1989; Gavrilets, 2015).

**FIGURE 1:**
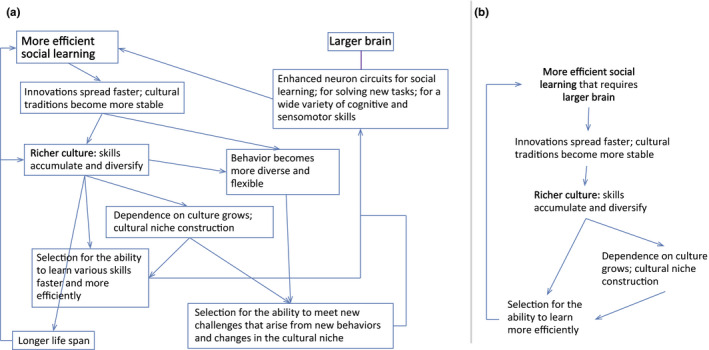
The logic of the positive feedback underlying the runaway brain‐culture coevolution, as suggested by the “cultural drive” hypothesis. (a) the logic of the cultural drive (based primarily on Laland, 2017), (b) minimalistic representation of the cultural drive, as implemented in TribeSim program

More recently, another type of hypothetical positive feedback mechanism termed “cultural drive” started to receive increasing attention among researches (Laland, 2017). The term was originally coined by Wilson (1985) who hypothesized that enhanced cognitive abilities, especially the abilities for social learning and cultural transmission of adaptive behaviors (Laland & Galef, 2009), can accelerate biological evolution. Smarter animals invent new adaptive behaviors more often and transmit them across generations more effectively; new cultural traditions create new selective environments (“cultural niche construction” [Laland & O'Brien, 2011; Laland, Odling‐Smee, & Feldman, 2001]) in response to which animals evolve faster (Wilson, 1985). The “cultural drive” (or “cultural brain”) hypothesis was further elaborated by Laland (2017) and other researches who suggested that the coevolution of social learning, cognitive abilities and culture can be self‐sustaining (Henrich, 2015; Heyes, 2012; Laland & Rendell, 2013; Muthukrishna, Doebeli, Chudek, & Henrich, 2018; Whiten, Ayala, Feldman, & Laland, 2017; Whiten & van Schaik, 2007). In its simplest form, the positive feedback mechanism of the “cultural drive” can be described as following: better social learning and cognition → more behavioral innovations become fixed as cultural traditions; richer culture → more skills available to be learned from conspecifics; increased usefulness of learning abilities; more sophisticated and flexible behavior results in new cognitive challenges → selection for still better social learning and cognition (Figure 1).

Additional positive feedback loops are conceivable, for example, via longer life span (elaborate culture → enhanced survival → longer life span → better prerequisites for intergenerational transfer of knowledge → still more elaborate culture → stronger selection for enhanced social learning) or via better nutrition (elaborate culture, including enhanced food acquisition strategies → enhanced nutrition → relaxed constraints for the evolution of larger brains) (Caspari & Lee, 2004; Crews, 2003; Kaplan & Robson, 2002; Laland, 2017; Muthukrishna et al., 2018).

The cultural drive hypothesis has received some theoretical (Henrich, 2015; Muthukrishna et al., 2018; Richerson & Boyd, 2005) and empirical support (Foote et al., 2016; Kopps et al., 2014; Whiten, 2017), including significant positive associations between brain volume (both absolute and relative) and social learning proclivity, longevity, social group size, and technical innovation in primates (Navarrete, Reader, Street, Whalen, & Laland, 2016; Reader & Laland, 2002; Street, Navarrete, Reader, & Laland, 2017) and cetaceans (Fox, Muthukrishna, & Shultz, 2017).

Importantly, the “cultural drive” hypothesis does not contradict other ideas such as “Machiavellian intelligence,” “social brain,” “cooperative brain,” “brain for stone tool production,” or “brain for mate attraction.” In fact, the “cultural drive” hypothesis can embrace a wide variety of such ideas, because it does not particularly rely on any specific type of culturally transmitted skills or behaviors. Potentially, any skills will do, given that they are cognitively demanding, require high‐fidelity social learning and provide reproductive benefits, thereby enhancing the spread of the good learners' genes.

This versatility is vividly illustrated by a computer model designed by Gavrilets and Vose (2006). Although their paper is titled “The dynamics of Machiavellian intelligence,” the model actually simulates cultural drive in one of its simplest forms. In fact, it accommodates the theories of Machiavellian intelligence and sexual selection within the framework of cultural drive. A brief description of this model is necessary for understanding the theoretical context of the current study. Simulated males in a polygynous, promiscuous population compete for mates. Males sporadically (and very rarely) invent “Machiavellian memes,” that is, behaviors that improve their competitive ability. Memes can be acquired by other males via social learning. The chances to successfully learn a meme depend on the meme size (“complexity”) and the male's memory capacity and learning ability. Both characters are “costly,” that is, they decrease survival (it is assumed that they require larger brains, although brain volume is not modeled explicitly). Genes for memory capacity and learning ability mutate at a specified rate. Meme size positively correlates with its fitness effect, but the correlation is weak. Initially, all males have zero memory capacity and learning ability. The evolution of the simulated population starts with a more or less prolonged “dormant phase” during which both memory capacity and learning ability remain low (as slightly deleterious traits at the mutation–selection equilibrium), and only newly invented memes are present in the meme pool. But sooner or later a self‐accelerating process starts which the authors call “cognitive explosion.” During this phase cognitive abilities, population's cultural richness (meme count) and Machiavellian fitness of individuals all increase in a runaway fashion. The cognitive explosion is fueled by cultural drive (although the authors do not use the term): The more memes there are in the meme pool, the more it pays to have good memory and learning ability (Gavrilets & Vose, 2006).

Here we elaborate on this approach by designing a more complicated model, TribeSim, aimed to explore the impact of different factors on the dynamics of the brain‐culture coevolution in a highly social species. The most important of the studied parameters include the extent of within‐ and between‐group competition, different types of memes (individually beneficial, group‐beneficial, useless, or maladaptive) and their combinations (specialized culture versus complex culture). We set out to elucidate the possible prerequisites for the most extensive brain expansion observed in the Pleistocene hominins, and we argue that the early representatives of the *Homo* clade, but not the other apes, probably found themselves in a situation suitable for an unprecedentedly powerful cultural drive.

The model presented here is broadly similar to the “Cultural Brain Hypothesis” (CBH) model designed by Muthukrishna and co‐authors (Muthukrishna et al., 2018). However, the two models differ significantly in many ways and explore different aspects and factors of the autocatalytic brain‐culture coevolution. We discuss the similarities and differences between the two models in a separate section.

## 2 TRIBESIM: DESCRIPTION OF THE MODEL

### 2.1 General principles

TribeSim is an individual‐based model designed to simulate genetic and cultural evolution in a population of a social species. Population consists of competing social groups. Maximum possible group size is specified by a parameter ***G***; after reaching this limit, the group splits in two halves. Group members engage in cooperative effort to acquire resources from the environment in competition with other groups. We dubbed it “collective hunting” (Stanford, 1999), although other behaviors, for example, collective defense of the group territory, can also be considered in this context.

The environment produces a fixed amount of resources per year (***R***). ***R*** indirectly determines the maximum possible population size. Average number of groups is determined by the combination of ***R*** and ***G***.

The resources acquired by the group are then distributed among the group members. Individuals compete with each other to increase their share. Thus, there are two levels of competition: between groups and between individuals, similar to the “nested tug‐of‐war” model which was previously used to show that between‐group competition is a powerful driver of within‐group cooperation (Reeve & Hölldobler, 2007).

The outcome of competition, and thus the amount of resources acquired by groups and individuals, depends on behavioral traits that can evolve genetically or culturally. In the current study, we focused primarily on two behavioral traits which we call “Hunting efficiency” (HE) and “Machiavellian trick efficiency” (TrE). Both traits depend on culturally transmitted skills (memes) which can be invented from scratch (with a fixed low probability) or acquired from other group members via social learning. Both traits can also evolve as genetically determined predispositions, but this option was not used in the current study. All individuals are born with genetically determined “starting” values of HE and TrE (10 and 0, respectively).

Higher values of HE benefit the group (HE is a “cooperative trait”), because the amount of resources acquired by the group is proportional to the sum of individual HEs of all group members who go hunting (“hunting effort” of the group) relative to other groups' hunting efforts. For instance, if ***R*** = 3,000 and the population consists of two groups with hunting efforts 2,000 and 4,000, then the groups will get 1,000 and 2,000 resources, respectively. The higher the hunting effort of a group, the less is the share of other groups. This results in intergroup competition which presumably was quite strong in the Pleistocene hominins (Henrich, 2015). If ***R*** exceeds the sum of the hunting efforts of all groups, then each group gets the amount of resources which is equal to its hunting effort. In this case, there is no between‐group competition. However, such situation is not likely to last long because the population, under any reasonable parameters that ensure basic survival and reproduction, quickly grows up to the carrying capacity of the environment, after which between‐group competition becomes inevitable.

Within groups, the resources are distributed according to the individual values of TrE. If all individuals have equal TrEs, the resources are divided equally. TrE is a “selfish trait”: high TrE benefits the individual but not the group. Importantly, the memory capacity of each individual is limited and costly (see below), so if one remembers many HE memes, less space is left for the TrE memes, and vice versa. This makes being a good hunter a somewhat altruistic strategy, while being a skillful trickster is “selfish.”

There are three levels of competition and selection.

1. *Group selection*. Between‐group competition for resources and selective survival, growth and “reproduction” (splitting) of the groups result in group selection which favors the development of “cooperative” traits (Darwin, 1874; Richerson et al., 2016).
2. *Individual selection*. Within‐group competition for a larger share of resources results in selective survival and reproduction of individuals. Individual selection favors the development of “selfish traits” which benefit the individual and are either deleterious or neutral for the group (TrE is mostly neutral for the group because enhanced reproduction of individuals with higher TrE compensates for poor reproduction of individuals with lower TrE, see below).
3. *Meme selection*. Memes compete for dominance in individual memory and in the group's meme pool (culture). With all other things being equal, meme selection favors memes that spread faster (those that are easier to learn or require less memory capacity to be remembered) (Gavrilets & Vose, 2006). The meme's fate may also be dependent on its influence on the individual and group phenotypes.

Individuals are diploid and reproduce sexually, with recombination. Genes are not linked; progeny receives one random copy of each gene from each parent. Phenotypic value of a genetically determined trait in a heterozygous individual is calculated as the mean of the “genotypic values” of the two alleles. Pairs are formed at random within groups (between‐group migration is a separate process); a pair produces one progeny if the parents have enough resources; both parents invest in progeny; pairs are formed anew each year.

Individuals perform the following types of actions:

1. *Machiavellian tricks* are performed by individuals with TrE > 0; tricks increase the share of the group resources received by the individual.
2. *Learning*: Acquisition of a meme from another (randomly chosen) group member, initiated by the learner. The probability of success depends on the presence of a meme known by another individual but not by the learner, meme size, free memory capacity of the learner, and phenotypic trait “Learning efficiency” (LE), which can evolve genetically and/or culturally. In the latter case, a special category of memes is added (LE memes).
3. *Teaching:* Active transfer of knowledge from teacher to learner. The probability of success, apart from the factors listed in the previous paragraph, depends on “Teaching efficiency” (TE) which also can evolve genetically and/or culturally (TE memes).
4. *Collective hunting* to obtain resources from the environment (Stanford, 1999).
5. *Useless actions* (e.g., ineffective and costly rituals); this behavior is guided by a special category of memes (“Useless memes”). This option is used to explore the effect of different factors on the spread of maladaptive cultural traits (Enquist & Ghirlanda, 2007).

Actions 4 and 5 are costly (resources are spent to perform them).

TribeSim can be used to simulate genetic and cultural evolution of several other traits, for example, ingenuity (chances to invent a new meme), propensity to learn, to teach, to participate in hunting (those who do not participate are “free riders”), and to apply costly punishment to free riders. Here, we describe only the options explored in the current study. Other parameters were set to constants, and mutation rates of the corresponding genes were set to zero.

The “evolvable” part of the genotype (genes that can mutate and evolve) in most of our experiments included only one gene which determines memory capacity (MC gene). In some experiments, we also made genes for LE and TE evolvable. We acknowledge that such traits are usually very polygenic; the simulated “genes” thus can be regarded as large linked sets of genes. However, the genetic details are not likely to significantly affect the main results discussed here.

Each copy of a gene has a “value” which is directly translated into phenotype (e.g., the starting value of MC gene is 0; thus, all individuals in the population initially have zero memory capacity; a heterozygous individual with two copies of MC gene with values 0 and 0.2 has MC = 0.1). MC can vary from 0 to infinity, LE and TE vary from 0 (zero chance to transfer a meme) to 1 (100% success rate). Mutations can be positive and negative: they either increase or decrease the value of the gene.

Genotypic values of MC, LE, and TE are linked to brain volume: increasing them results in larger brains. This is in concordance with empirical correlations between brain volume and social learning in primates (Reader & Laland, 2002; Street et al., 2017), cetaceans (Fox et al., 2017), and presumably birds (van Schaik, Isler, & Burkart, 2012). Brain volume is a costly trait because the amount of resources needed to produce a child is proportional to the child's brain volume. This agrees with the idea that parental investment increased greatly in the course of hominin evolution along with the energetic and cognitive demands of the rapidly growing juvenile brain (Hublin, Neubauer, & Gunz, 2015; Leigh, 2012).

Memes are stored in memory and affect behavioral phenotypic traits (HE, TrE, LE, TE, and probability of performing a useless action). Memes are rarely invented (like in Gavrilets & Vose, 2006), can be transferred via social learning and forgotten. Each meme is unique; only one copy of each meme can be stored in individual memory. Each meme is characterized by its category (HE, TrE, etc.), size (or complexity; the amount of MC needed to store the meme), and efficiency (the increase in the phenotypic trait of an individual who knows the meme). Size and efficiency are positively correlated, but the correlation is weak (like in Gavrilets & Vose, 2006). Large memes can only be learned by individuals with sufficient free MC; thus, meme size is limiting its propagation, and MC limits the learning potential of an individual. LE affects the probability of successful meme transfer regardless of the meme size (the reasons for this are discussed below). The chance to forget a meme is fixed (2% per year for all memes and all individuals).

The individual phenotype includes the following variable traits:

1. *Propensity to perform useless actions.* The trait is calculated as the sum of probabilities defined by the efficiencies of Useless memes. For example, if an individual knows two Useless memes with efficiencies 0.2 and 0.3, then the propensity to perform useless actions is 0.2 + (1–0.2)*0.3 = 0.44. This means that the individual will perform useless actions with probability 0.44 per year. The cost of useless actions is fixed (1 resource is spent for each action).
2. *Hunting efficiency (HE)* is calculated as the sum of a fixed genetic value (HE gene was set to 10, mutation rate to zero, in all experiments) and the efficiencies of all HE memes known by the individual. For example, if an individual knows two HE memes with efficiencies 0.9 and 3.4, then HE = 10 + 0.9 + 3.4 = 14.3.
3. *Machiavellian trick efficiency (TrE)* is the sum of the efficiencies of TrE memes (TrE gene was set to zero and did not mutate). Like HE, it may vary from 0 to infinity.
4. *Learning efficiency (LE)* is the probability of successfully learning a meme. It may vary from 0 to 1. It is calculated as the sum of probabilities defined by LE gene and LE memes. LE gene mutation rate (if not set to zero) is 0.04 per gamete, mutation effect (change in genetically defined LE value) is normally distributed around zero with standard deviation 0.4. If the resulting value of the gene is negative or exceeds 1, mutation is canceled, and attempt is repeated. LE is broadly analogous to the social learning fidelity which is thought to be essential for the brain‐culture coevolution (Laland, 2017; Lewis & Laland, 2012; Muthukrishna et al., 2018).
5. *Teaching efficiency (TE)* is the probability of successfully teaching a meme to a group mate. It is calculated in the same way as LE. In most experiments, TE gene was set to 0, its mutation rate was 0, and TE memes were not allowed. Alternatively, TE gene mutated in the same way as LE gene (see above).
6. *Memory capacity (MC)* is genetically defined and can vary from 0 to infinity. Initial value of MC gene is 0, mutation rate 0.04 per gamete, mutation effect (change in MC value) is normally distributed around zero with standard deviation 0.4. Free memory capacity is MC minus the sum of the sizes of all memes kept in memory.
7. *Brain volume* is equal to 20 + k_1_MC + k_2_LE_g_ + k_3_TE_g_, where LE_g_ and TE_g_ are genotypic values of LE and TE. By default, the parameters k_1_, k_2_, and k_3_ are 1, 0, and 0.

The default set of parameters is described in more detail in Table S1.

### 2.2 Succession of events during one step of the simulation

The life of the simulated population consists of steps (years). The following events take place every year.

1. *Spending resources on life support:* 3 resources per year are taken from each individual.
2. *Spontaneous invention of new memes.* An individual invents a meme of a given category with a fixed, low probability (default value is 0.000133 per year), regardless of the number of meme categories allowed.
3. *Spontaneous forgetting of memes.* Each individual can forget any meme with probability 0.02 per year.
4. *Teaching.* Each individual randomly selects a group mate and tries to teach him or her one meme. The meme is selected at random from those known by the teacher but not by the student. If there are no such memes, the attempt fails. If the selected meme is larger than the student's free MC, the attempt fails. Otherwise, the probability of success is the sum of probabilities defined by the teacher's TE and the student's LE.
5. *Collective hunting* (Stanford, 1999). All individuals who possess enough resources go hunting; the cost of the action is 2 resources. For each group, its hunting effort is calculated as the sum of hunting efficiencies (HEs) of the hunters. If the sum of the hunting efforts of all groups is less than 3,000 (***R***, the amount of resources supplied by the environment per year), then each group receives the amount of resources which is equal to its hunting effort. Otherwise, each group receives its share of 3,000 resources which is proportional to the group's hunting effort.
6. *Sharing the resources.* By default, the resources obtained by the group are shared equally among all group members. Such egalitarianism is reminiscent of the traditional behavior of some hunter–gatherers (Hawkes, O'Connell, & Blurton Jones, 2001), and even chimpanzees often share meat after successful hunt (Gilby, 2006). However, if there are individuals with TrE > 0, they perform “Machiavellian tricks” to claim larger share. The resources are then distributed according to the individual values of TrE. Consequently, for a naive (e.g., young) individual it is difficult to survive among skillful tricksters, unless she quickly learns those memes herself.
7. *Useless actions.* If an individual knows Useless memes, he performs a useless action with probability calculated as the sum of efficiencies of these memes. The cost of a useless action is 1 resource.
8. *Learning*. Each individual randomly selects a group mate and tries to learn a meme from her. The meme is selected randomly from the memes known by the potential teacher but not by the student. If there are no such memes, or if the size of the selected meme exceeds the free MC of the student, the attempt fails. Otherwise, the probability of success equals to the student's LE.
9. *Death.* The probability of death depends on the individual's age. By default, it is equal to age multiplied by 0.002. This results in average life span of about 27 years. Additionally, an individual can die of hunger if he or she does not have enough resources for life support for two years in a row (one hungry year often follows the birth of a child and is not lethal, see below). If an individual does not have enough resources to perform a costly action (e.g., hunting or a useless action), then the action is not performed. If there is only one individual left in the group, he dies.
10. *Reproduction.* Each individual older than 6 years attempts to form a pair with a group mate and produce a child. Pairs are formed for one year only (serial monogamy). If there are no unpaired individuals in the group, the attempt fails. After the pair is formed, the possibility of producing a child is tested. To produce a child, the parents have to spend the amount of resources which is equal to the proposed child's brain volume multiplied by 2. 40% of these resources are transferred to the child. If both parents together do not have enough resources, the attempt to produce a child fails. After the child is produced, and if the parents have some resources left, 40% of them are also transferred to the child, and the remainder is distributed equally among the parents. The equality of parents in TribeSim is reminiscent of the supposedly increased paternal care, decreased sexual dimorphism and trend toward monogamy and cooperative breeding in hominins (Kramer & Russell, 2015; Lovejoy, 2009; Stanyon & Bigoni, 2014). For simplicity, the simulated individuals in TribeSim do not have a fixed gender; any two individuals can form a pair and produce offspring.

The child inherits one randomly chosen copy of each gene from each parent. Genes for MC (and sometimes also LE and TE) can mutate and therefore evolve (see above). Mutations occur when the genes are passed from parent to child. The child's memory is initially empty.

- *Splitting of the groups.* If the group exceeds its upper limit ***G***, it splits in two equal groups.
- *Between‐group migration.* An individual can leave her group and join another (randomly selected) group with a specified probability (0.001 per year by default).

The parameter values were selected arbitrarily based on the general logic of the model (e.g., new memes are rarely invented and can be highly useful; reproduction costs rapidly increase with brain volume, etc.) and the experience of the preliminary model runs (e.g., the genetic value of “Hunting efficiency” was set to 10 to ensure that the population can survive and quickly grow to the carrying capacity of the environment even in the absence of adaptive knowledge). We had to limit the resources provided by the environment so that the population size would stay within computationally tractable limits. For the same reason, we sought to ensure that the evolving population would approach equilibrium state within 70,000 years, which is apparently much less than the real period of brain expansion in human evolution (about two million years). High gene mutation rate and highly variable (and sometimes very strong) phenotypic effects of memes are the measures we took to accelerate evolution. Although we tried to set most of the parameters within the limits that seem broadly realistic, no attempt was made to simulate any real primate species or to obtain quantitatively precise predictions. We discuss the effects of variation of the most important parameters in a separate section.

## 3 RESULTS AND DISCUSSION

For the sake of clarity, we shall first describe the results of the most simple simulations wherein most parameters were fixed, most genes could not mutate and evolve, and only two types of memes were allowed. Later on, we shall gradually add new degrees of freedom and explore new dimensions of the parameter space.

### 3.1 Cultural drive in an undivided population with “Machiavellian” culture

First, we explored the dynamics of the simulated population under the following very simple conditions: all population is a single group (no between‐group competition), only TrE and Useless memes are allowed, social learning is limited only by MC, brain volume = 20 + MC (which means that parents have to spend two additional resources for each additional unit of the child's memory capacity), initial MC = 0, MC gene is the only gene that can mutate and therefore evolve, LE = 1 and does not evolve (perfect congenital copying ability), TE = 0 (see Table S1 for further details).

Under such parameters, population stabilizes at 450–500 individuals, a new meme is invented approximately each 5–6 years. Population is quite viable from the start and quickly reaches the carrying capacity of the environment. There is no need for change, but the changes are forced by within‐group competition and “cultural drive.” As soon as any individual invents and remembers an efficient TrE meme, she is at advantage. One needs MC to learn the meme, and so selection for MC starts. The process is autocatalytic: the higher is the average MC, the higher is the probability of preservation and spread of newly invented memes. The larger is the number of useful memes in the meme pool, the more beneficial it is to have large MC. As group members become more sophisticated tricksters, it becomes harder for individuals with low MC (and small brain) to survive and reproduce in such a group. This is an example of the increased “dependence on culture” (Figure 1).

The runaway brain‐culture coevolution forced by cultural drive is reminiscent of Baron Munchausen pulling himself out of a swamp by his hair: There are no external incentives and no reason to change, but the growing brain and developing culture continue to push each other further and further.

**FIGURE 2:**
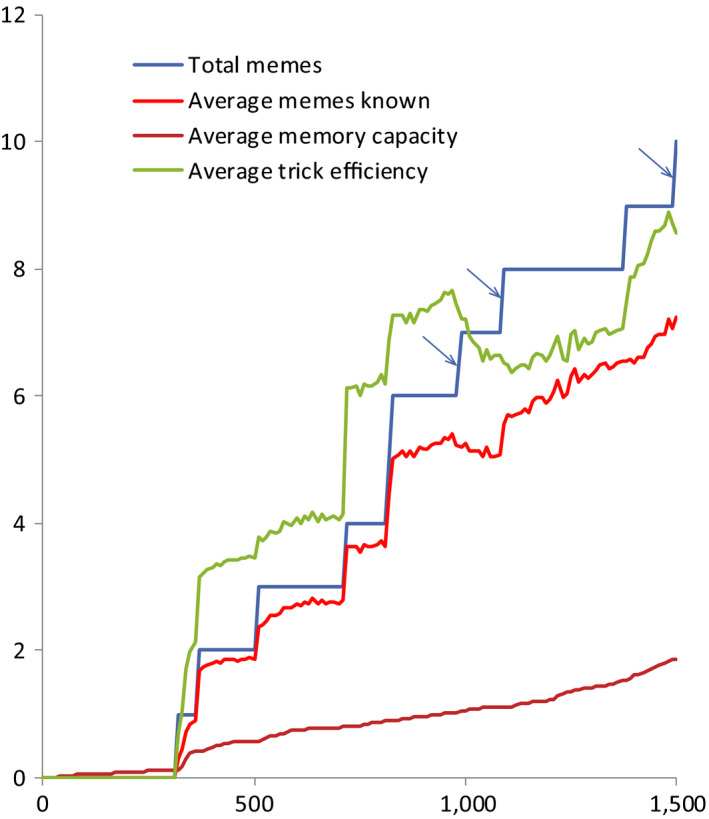
Initial stages of brain‐culture coevolution under basic parameters (no between‐group competition, “Machiavellian” culture, see Table S1). See text for further explanations

The initial stages (first 1,500 years) of brain‐culture coevolution under these parameters are shown in Figure 2. At first, there is a “dormant phase”: No memes are preserved because individuals do not have enough MC. Initially, MC = 0, but MC gene mutates, and mutant individuals with MC > 0 appear. As long as there are no useful memes in the meme pool, MC is a slightly deleterious trait (more resources are required to produce offspring). This is in concordance with the notion that social learning is maladaptive in a world with little knowledge (Muthukrishna et al., 2018). Thus, the average MC tends to mutation–selection equilibrium which in this case corresponds to MC_avg_ ≈ 0.3. Average size of invented memes is 1, but the variance is high. So sooner or later a small enough meme is invented by an individual brainy enough to remember it. Efficient TrE meme can promote selection for MC and thus initiate the “cultural drive.”

Useless memes cannot initiate cultural drive. Moreover, if the first meme to spread happens to be a small but efficient Useless meme, then large MC may become considerably more deleterious, and mutation–selection balance may shift to a lower equilibrium value of MC. This can prolong the “dormant phase.” As the cultural drive proceeds, Useless memes infiltrate the meme pool (in the example shown in Figure 2, three Useless memes have spread, marked by arrows). However, under the current set of parameters parasitic memes cannot stop cultural drive. Littering of memory by Useless memes results in continued brain/memory expansion so that it can accommodate TrE memes despite being littered.

**FIGURE 3:**
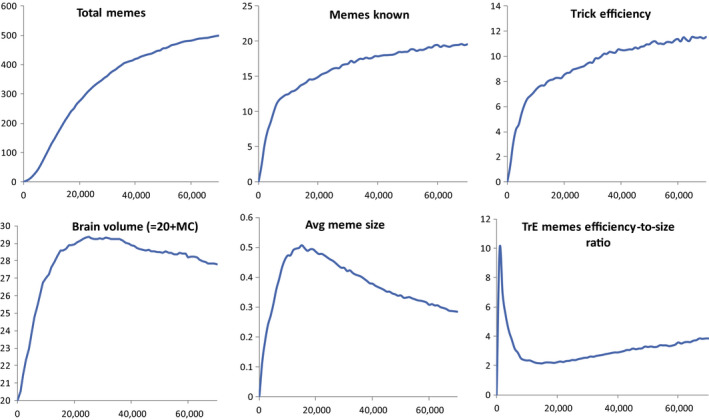
Brain‐culture coevolution under basic parameters (no between‐group competition, “Machiavellian” culture, see Table S1). Averages from 10 model runs are shown

Further events (up to the year 70,000) are shown in Figure 3.

Cultural richness (total number of unique memes in the meme pool) follows an S‐shaped curve. Average number of memes known by an individual decelerates somewhat faster because this parameter is limited by life span and learning speed. Average individual tends to know only about 4% of the total knowledge of the population.

Average brain volume grows driven by more and more efficient Machiavellian culture, but then it stops growing and even declines. The limit to growth is explained by the fact that at some point the benefit from the ability to learn more TrE memes stops to exceed the cost of further brain expansion. The cost grows linearly with brain volume (the larger the brain, the more resources are spent by parents for each child). The benefit, however, increases with deceleration, because it takes time to fill a large memory. As culture and brain expand, the “juvenile” period of incomplete use of memory becomes longer. This is in concordance with the empirical positive relationship between the length of the juvenile period and brain size in primates (Joffe, 1997; Street et al., 2017; Walker, Burger, Wagner, & Von Rueden, 2006). As individuals start to fully exploit their large memory at progressively later age, the benefit from further brain/memory expansion decreases. At some point, the benefit stops to exceed the cost, and the brain stops growing.

The reasons for further decline in brain volume are more subtle. They stem from the gradual decrease in the average size of memes stored in memory (Figure 3), a process observed under virtually any parameters, unless we make the sizes of all invented memes equal. This process, which we call “meme simplification,” is driven by meme selection: smaller memes spread faster irrespective of their phenotypic effects, because there are always individuals in the population whose free MC is insufficient to learn a large meme, but is still large enough to learn a small meme. Meme simplification was observed by Gavrilets and Vose in their model (Gavrilets & Vose, 2006) and is clearly reproduced in our study.

For the first ~15,000 years, the average size of the memes stored in individuals' memories was increasing (Figure 3). This is because meme's size is positively correlated with its efficiency, and average MC was growing rapidly, thus making it possible to remember larger (and, on average, more useful) TrE memes. Later, however, selection for smaller memes led to meme simplification. Accumulation of simple (small) memes in culture makes large MC less beneficial, because learning takes time, and at some point individual life spans become insufficient to fill large memory with progressively smaller memes. The efficiency of memory usage decreases, and the brain starts to shrink.

The integral efficiency of Machiavellian culture (average phenotypic TrE of individuals, Figure 3) continues to grow despite the decrease in MC, because memes not only become smaller: They also become relatively more efficient (efficiency‐to‐size ratio increases) due to the interplay of meme and individual selection.

**FIGURE 4:**
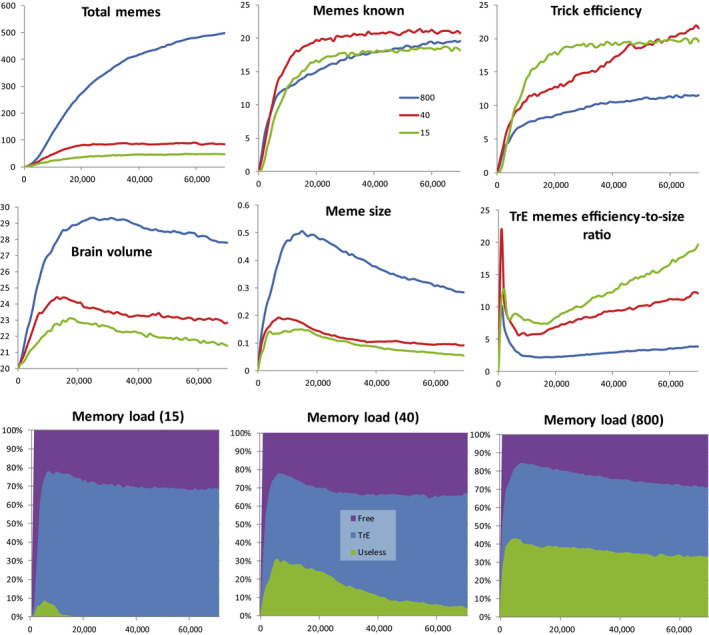
Brain‐culture coevolution under three levels of between‐group competition: no competition (G = 800), moderate competition (G = 40), and strong competition (G = 15). The culture is “Machiavellian,” all other parameters as in Figure 3 and Table S1. Averages from 10 model runs are shown

Interestingly, the proportion of memory occupied by Useless memes decreases only slightly with evolutionary time, and the proportion occupied by TrE memes does not increase (Figure 4, bottom right diagram), although TrE memes are highly beneficial and Useless memes are slightly deleterious. This reflects the fact that individual selection has only a very limited ability to remove maladaptive memes from the culture (Enquist & Ghirlanda, 2007) or, more generally, to control the content of the meme pool. This is because memes, unlike genes, spread horizontally. If a maladaptive meme does not kill its host immediately, which Useless memes do not, the host has time to disseminate the meme. Conversely, between‐group competition and cultural group selection (Richerson et al., 2016) can purge the culture very efficiently (see below).

The evolution of the simulated population can be divided into three stages (observed under other sets of parameters as well).

1. Start of the runaway brain‐culture coevolution: “rough” culture stimulates rapid brain expansion. At first, the brain starts to expand to accommodate the first small but efficient memes. As the brain grows, the average size of the memes also grows, while their efficiency‐to‐size ratio decreases (“extensive” development of culture). The culture formed during this stage is “rough”: It consists of large memes with low efficiency‐to‐size ratio. It is this culture, however, that stimulates the fastest expansion of the brain.
2. The potential for “extensive” development is exhausted. Meme selection results in gradual meme simplification. As memes become smaller, brain expansion slows down and stops; this results in further selection for smaller memes. The integral efficiency of Machiavellian culture continues to increase, as seen from the increase in average phenotypic TrE of individuals. The efficiency‐to‐size ratio of the memes starts to increase again.
3. “Intensive” cultural development. Further meme simplification promotes the decrease in brain volume. “Childhood” (the period needed to fill one's memory with memes) becomes longer. The integral efficiency of culture is still growing; the culture becomes more “sophisticated.”

Perhaps it is not too far‐fetched to note that the three stages are somewhat reminiscent of the Early, Middle, and Late Paleolithic, although there are prominent differences as well. Most importantly, in our model the cultural development is generally decelerating (rather than accelerating as in the real history of *Homo*), and the relative durations of the stages are reversed. We believe that the discrepancies stem from the fact that the simulated culture is not cumulative: Memes cannot be modified, improved, or used as the basis for further development. Modeling cumulative culture is a task beyond the scope of the current study.

### 3.2 Cultural drive in a population consisting of competing groups; “Machiavellian” culture

Next, we asked how between‐group competition (BGC) influences the brain‐culture coevolution. We used the same set of parameters with one exception: We varied the value of ***G*** (maximum group size), which was initially set to 800 so that the population always consisted of a single group. We tried two other values: 40 and 15, which correspond to approximately 20–25 and 55–65 groups in the population, respectively. This results in moderate to strong BGC and group selection.

The results are shown in Figure 4. BGC alters the pattern of brain‐culture coevolution. The outcome of BGC generally depends on cultural differences between groups (cultural group selection [Richerson et al., 2016]) and on brain volume (large brain hinders reproduction and group propagation). In this case, culture per se is useless for competition with other groups, because only TrE and Useless memes are allowed. TrE memes only influence the distribution of resources within the group. This results in “reproductive skew” (Muthukrishna et al., 2018): knowledgeable individuals, who are generally older, reproduce better than “naive” individuals who are generally younger. Machiavellian culture has no significant direct impact on the competitive ability and propagation of the group, because slower reproduction of some individuals is compensated by faster reproduction of the others. However, despite their inability to improve the group's competitive ability, TrE memes are still capable of initiating cultural drive. In a way, Machiavellian culture harms the group in the long term by enforcing brain expansion which, in turn, results in reduced fecundity. Those groups that can somehow curb cultural drive will be at advantage. As seen from Figure 4, what really happens is that BGC promotes stronger selection for smaller memes and effective purging of culture from Useless memes. Both processes help the brain to remain small despite the cultural drive.

BGC results in lower cultural richness (Figure 4), mostly because of cultural drift: groups compete, often die out (with their culture), or split and propagate. This results in lower overall meme diversity. However, average individual knows approximately the same number of memes as in the previous case (without BGC). Overall, the culture is less diverse and more stereotypic.

**FIGURE 5:**
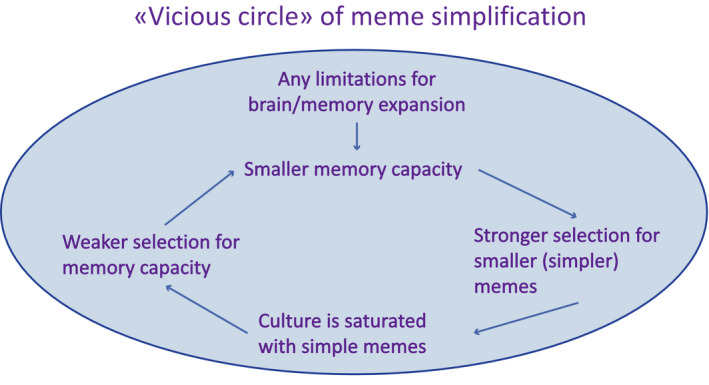
“Vicious circle of meme simplification.” The necessary assumption is that learning one meme of the size *n* takes less time than learning *m* memes of the size *n/m*, which seems likely. Under such circumstances, the saturation of culture with smaller memes results in weaker selection for memory capacity due to less efficient lifetime‐wise use of memory

BGC also results in lower MC and brain volume. When there is no BGC (***G*** = 800), only individual selection is working against brain expansion. When BGC is present, individual selection is aided by group selection. Consequently, the limitations on brain expansion become more stringent. This, in turn, results in stronger selection for smaller memes, more radical “meme simplification” and higher efficiency‐to‐size ratio for TrE memes. Smaller memes, once again, reduce selection for larger brains. We call this feedback mechanism “the vicious circle of meme simplification” (Figure 5).

The integral efficiency of Machiavellian culture is, somewhat unexpectedly, even higher than without BGC. This is because TrE memes which accumulate in the meme pool are extremely small and efficient, and Useless memes are effectively removed. BGC cannot stop “selfish” brain‐culture coevolution driven by the TrE memes, but it is fully capable of removing Useless memes from the culture. Groups with additional costly MC filled by maladaptive memes loose the competition. Expectedly, such efficient cultural group selection is possible only if between‐group migration rate is comparatively low (in the simulations discussed so far, individuals move to another group with probability 0.001 per year; this means that only 2%–3% of individuals migrate in their lifetime). The higher the migration rate, the more similar is the pattern of brain‐culture coevolution to that observed in the absence of BGC (see below).

### 3.3 “Machiavellian” culture versus “cooperative” culture

Next, we simulated brain‐culture coevolution in a population with “cooperative” (rather than “Machiavellian”) culture. The same parameters were used as before, with the only exception that “Hunting efficiency” (HE) memes were allowed instead of TrE memes. The parameters for HE memes (mean efficiency, standard deviation of efficiency, C, R [see Table S1 for explanations]) were 4, 6, 0.25, and 2. This means that an average HE meme increases the phenotypic value of HE by 4 units, has size 1, there is a weak positive correlation between the efficiency and size, but the variance of both values is high. The situation is thus completely symmetrical to the previous one, except that TrE memes are beneficial for the individual but useless for the group, while HE memes are beneficial for the group rather than for the individual. The cost of having larger brains is paid by the individual, while the benefit from remembering more HE memes is shared between all group members.

**FIGURE 6:**
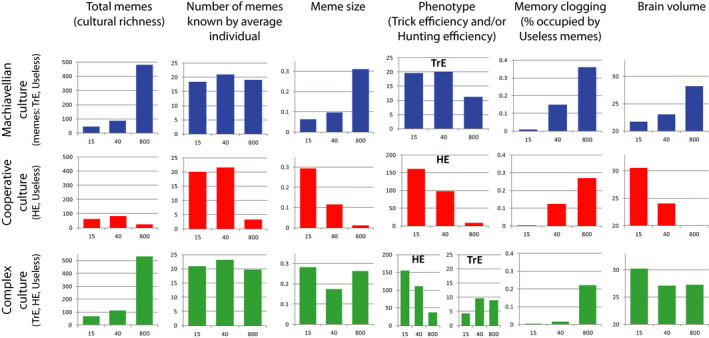
Simulation results for three different cultures (Machiavellian, Cooperative, Complex) and three levels of between‐group competition (15—very strong, 40—moderate, 800—absent). Averages from 10 model runs are shown. For Memory clogging, averages are shown for years 1–70,000; for other parameters, averages are shown for years 50,000–70,000

The results are shown in Figure 6 (middle row of diagrams). For comparison, simulation results for Machiavellian culture (discussed above) are shown in the top row of diagrams.

When brain expansion is driven by cooperative culture, the intensity of BGC is the major factor promoting cultural drive. Contrary to Machiavellian culture that provides maximum brain expansion under minimal BGC, cooperative culture results in larger brains under strong BGC. If BGC is absent (***G*** = 800, all population is a single group) or low (***G*** = 300, population consists of 3–5 groups, data not shown) cooperative culture fails to initiate cultural drive, and the brain remains small. Thus Machiavellian culture appears to be able to initiate cultural drive under a wider range of BGC levels than cooperative culture.

If BGC is strong (***G*** = 15), cooperative cultural drive is very efficient, and brain volume reaches even higher values than in the case of Machiavellian culture and no BGC. Moreover, under strong BGC and cooperative culture, both meme simplification and decrease in brain size are less pronounced and start later. This is because every HE meme, regardless of its size, is very important for group survival when competition with other groups is high. Cultural group selection strongly favors groups with the most efficient cooperative (hunting) culture. The resulting culture is “rough” and uniform on the population scale, but very efficient. The results are in accord with the idea that cultural group selection was essential for the evolution of human cooperation (Darwin, 1874; Richerson et al., 2016).

BGC appears to be a powerful mechanism to prevent the spread of the Useless memes. This is true for cooperative culture as well as for Machiavellian culture. Strong BGC generally results in a uniform, efficient culture with a minimum amount of unnecessary elements.

The comparison of the top and the middle rows of diagrams in Figure 6 shows that the average meme size covaries with the average brain volume (MC). This is apparently because smaller brains (or stronger constraints on brain expansion) promote stronger selection for smaller memes, and saturation of culture with smaller memes results in weaker selection for larger brains (Figure 5).

### 3.4 Complex culture versus specialized culture

Next, we simulated brain‐culture coevolution in a population with a complex culture, that is, with all three types of memes allowed (TrE, HE, Useless). All parameters were as in previous simulations, except that creativity was set to 0.0004, in order to retain the fixed rate of invention of memes of each type (0.000133 per individual per year, see Table S1).

**FIGURE 7:**
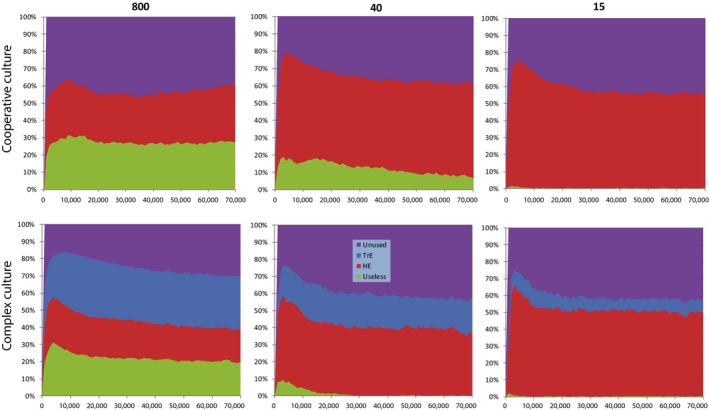
The dynamics of memory loading for two cultures (cooperative and complex) and three levels of BGC (15—very strong, 40—moderate, 800—absent). Averages from 10 model runs are shown. The left top diagram (Cooperative culture, G = 800) depicts the situation when the runaway brain‐culture coevolution did not start, brain and MC remained very small, and the culture consisted of very few simple memes

The results are shown in Figure 6 (bottom row of diagrams). The complex culture appears to be a more powerful driver of the runaway brain‐culture coevolution than both types of specialized culture discussed above. The differences are as follows:

1. The complex culture promotes strong cultural development and brain expansion at all levels of BGC (contrary to Machiavellian culture which works best at low levels of BGC, and to cooperative culture which initiates cultural drive only at sufficiently high levels of BGC);
2. The complex culture results in a slightly richer meme pool and slightly lower levels of memory clogging by the Useless memes. Under strong BGC, “cooperative” (group‐beneficial) HE memes outcompete “selfish” (individually beneficial) TrE memes (Figure 7, bottom row of diagrams).
3. The complex culture makes it possible for some hunting skills to develop even when HE memes are not supported by group selection. For example, the average phenotypic value of HE under complex culture and ***G*** = 800 is much higher than under specialized cooperative (hunting) culture and ***G*** = 800. When ***G*** = 800, there is no BGC and the HE memes are virtually useless, because the initial (congenital) phenotypic value of HE is high enough for the individuals to survive and reproduce. The population quickly grows up to the carrying capacity of the environment, after which all available resources are effectively extracted and used. Thus, in the absence of BGC the HE memes provide no benefits to either individuals or the group. This is why cooperative culture is unable to initiate runaway brain‐culture coevolution in the absence of BGC. However, when ***G*** = 800 and the culture is complex, cultural drive is initiated by the TrE memes, the brain and MC expand, and the HE memes begin to spread as “parasitic” memes despite their uselessness under the circumstances.
4. The complex culture prevents the extreme meme simplification.

The dynamics of memory loading (Figure 7) shows that cultural group selection (which is efficient when BGC is high) is capable of shaping the content of the meme pool, while individual selection is not. When ***G*** = 800, the proportions of the meme types stored in memory are similar to the proportions of meme types invented, whereas under ***G*** = 15 cooperative HE memes prevail in the meme pool. This is in accord with the ideas that culture can evolve in a Darwinian fashion, that intergroup competition shapes cultural evolution, and that cultural group selection was an important driver of the evolution of human cooperation (Henrich, 2015; Richerson et al., 2016).

The advantages of the complex culture discussed above imply that the chances for a large‐scale runaway brain‐culture coevolution may improve when the population finds itself in a situation where the individuals have the opportunity to invent many different, cognitively demanding, and highly beneficial memes of different types, for example, cooperative *and* Machiavellian. In this case, cultural drive is possible irrespective of the intensity of BGC.

This situation is conceivable for early *Homo.* Chances to invent valuable and cognitively demanding “cooperative” memes could have increased due to changes in foraging behavior and new feeding strategies, for example, cooperative scavenging or hunting for large prey in savannah habitats (Braun et al., 2010; Patterson et al., 2019; Rogers, Harris, & Feibel, 1994; Stanford, 1999). Early hominin scavengers and hunters are thought to have relied heavily on within‐group cooperation in order to effectively compete with large carnivores and other hominins (Bickerton & Szathmáry, 2011; Flinn, Geary, & Ward, 2005; Gavrilets, 2015; Henrich, 2015; Moll & Tomasello, 2007). Stone tool production and use is an example of behavior which probably was highly beneficial for the group because fast and effective butchering of large carcasses, along with other types of cooperative behavior, could have been essential for avoiding direct aggressive encounters with stronger competitors and predators (Plummer, 2004; Rose & Marshall, 1996). At the same time, even the production of seemingly simple and primitive Oldowan tools appears to be cognitively demanding and requiring high‐fidelity social learning (Morgan et al., 2015). We suggest that socially transmitted skills needed for stone tool production and use by early *Homo* may be comparable with the “Hunting efficiency” memes in our simulation. Confrontational scavenging, hunting, and butchering were most probably collective endeavors whose success benefited the group rather than an individual hunter or butcherer.

Chances to invent highly beneficial and cognitively demanding “Machiavellian” memes could have increased due to the proposed evolutionary shift toward lower within‐group aggression, social monogamy, cooperative breeding, increased social conformity, parental care, and paternal investment in offspring (Lovejoy, 2009; Raghanti et al., 2018; Stanyon & Bigoni, 2014). In a society where direct physical aggression against group members is not encouraged, “Machiavellian” skills and tricks may become the main way to achieve higher status and reproductive success (Byrne & Whiten, 1988; Humphrey, 1976).

### 3.5 High‐fidelity, costly social learning is a powerful driver of brain expansion

Selection for high‐fidelity social learning is the cornerstone of the “cultural drive” hypothesis (Laland, 2017; Lewis & Laland, 2012; Muthukrishna et al., 2018). In the simulation experiments described above, social learning was limited by memory capacity which was costly in terms of brain expansion (each unit of MC required one additional unit of brain volume). This cost is nearly optimal for achieving the largest brain size: Under the current parameters, it is not possible to achieve much larger brain volumes by making it higher or lower. If the cost is lower, the extent of cultural development ultimately will be similar (limited in the long term primarily by learning speed and life span), but the brain will be smaller. With higher cost, the “vicious circle of meme simplification” will be more intense, the average meme smaller, and selection for larger brains weaker. The resulting culture will again be similar, and the brain volume smaller.

In an attempt to find additional factors promoting brain expansion, we varied the parameter LE (“learning efficiency”) which determines the probability of success when an individual with sufficient MC attempts to learn a meme. In the experiments discussed above, LE was set to maximum (LE = 1), did not evolve, and did not impose any costs. In the next experiment, we set the initial value of LE to 0, made it very costly (30 units of brain volume per unit of LE), and allowed it to evolve genetically (LE gene mutation rate 0.04, mean mutation effect 0, standard deviation 0.1). All other parameters were left unchanged. This means that brain‐culture coevolution was now limited not only by the costly memory capacity, but also by the very costly learning efficiency. In other words, much higher price must be paid now for the same level of cultural development.

This is different from just making MC more costly, because there is one major difference between MC and LE as the limiting factors of brain‐culture coevolution in our model. Costly MC limits the spread of large (complex) memes to a much larger extent than the spread of smaller memes, thus providing selective advantage to simple memes and facilitating “the vicious circle of meme simplification.” Conversely, costly LE limits the spread of all memes irrespective of their size (complexity). Thus, costly LE is not expected to promote meme simplification. Of course, LE can also be programmed to select for smaller memes (as was done by Gavrilets and Vose (2006)), but we aimed to explore a complexity‐insensitive limiting factor (LE) along with a complexity‐sensitive one (MC).

**FIGURE 8:**
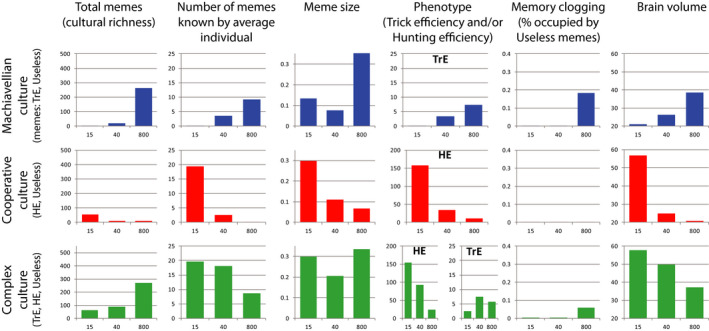
Brain‐culture coevolution with evolvable, costly learning efficiency (see text for explanation). All other parameters and designations as in Figure 6. Averages from 10 model runs are shown. For Memory clogging, averages are shown for years 1–70,000; for other parameters, averages are shown for years 50,000–70,000

Rather unexpectedly, we found that very costly LE does not prevent the runaway brain‐culture coevolution. Under some combinations of parameters ([complex culture, ***G*** = 15] and [cooperative culture, ***G*** = 15]), average LE evolves up to 0.89–0.91 within 70,000 years despite the fact that the cost of such evolution is 26.7–27.3 additional units of brain volume. The results are summarized in Figure 8 (compare with Figure 6 to see the effects of evolvable, costly LE; note different scales on the brain volume diagrams).

Costly, evolvable LE (Figure 8) results in cultural development similar to that observed under free, fixed LE (Figure 6). Runaway brain‐culture coevolution starts under all combinations of parameters except [cooperative culture, ***G*** = 800] and [Machiavellian culture, ***G*** = 15]; under [Machiavellian culture, ***G*** = 40] it starts in approximately half of model runs. Total cultural richness is somewhat lower than in the case of free, fixed LE, but the integral efficiency of the resulting culture (phenotypic values of TrE and HE) are comparable. Meme simplification is slightly less pronounced (average meme size is larger). Parasitic memes are more efficiently removed from the meme pool. The most striking differences are in the brain volume: When LE is costly and evolvable, brain volume reaches much higher values. Brain expansion is especially pronounced when the culture is cooperative or complex and between‐group competition is high. This is in concordance with other theoretical studies that addressed possible positive effects of higher between‐group competition (or relatively lower within‐group competition) on human brain expansion (Gavrilets, 2015; González‐Forero & Gardner, 2018; Muthukrishna et al., 2018).

Why does the costly learning efficiency result in evolution of larger brains than the costly memory capacity? The apparent reason is that LE is insensitive to meme complexity, and so the constraints on LE do not result in the “vicious circle of meme simplification,” whereas the constraints on MC do.

The results imply that the runaway brain‐culture coevolution can be strongly facilitated by the development of mechanisms of social learning that are (a) costly in terms of brain expansion or structural optimization and (b) tolerant to the complexity of knowledge. In other words, these mechanisms must be neurologically demanding (e.g., rely on sophisticated neuronal circuits that span many different brain areas), and they must make it possible to transfer complex information relatively easily. Human language seems to fit this definition accurately (e.g., Lieberman, 2002).

**FIGURE 9:**
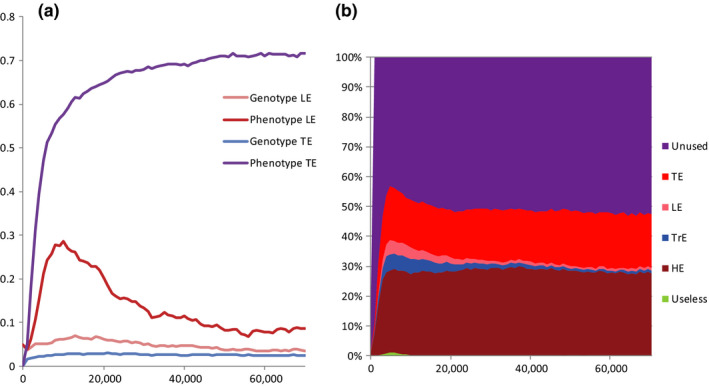
Evolution of social learning (LE, TE). (a) genotypic and phenotypic values of LE and TE (phenotypic value = genotypic value + sum of the effects of the corresponding memes). The efficiency of “learning culture” or “teaching culture” is phenotype minus genotype. Note the extensive cultural evolution of teaching efficiency. (b) memory loading; note that the TE memes occupy much larger proportion of memory than LE and TrE memes. Only HE memes are more successful, because they are strongly favored by the cultural group selection. Averages from 10 model runs are shown. Parameters: G = 40 (moderate between‐group competition); 5 categories of memes are allowed (TrE, HE, Useless, LE and TE); parameters for LE and TE memes: mean efficiency 0.2, standard deviation of efficiency 0.3; C = 5; R = 2 (see Table S1 for further explanations); genes for MC, LE, and TE can mutate (for LE and TE genes: mutation rate 0.04, mean mutation effect 0, standard deviation 0.1); brain volume = 20 + MC + 30 × (genotypic value of LE + genotypic value of TE); initial values of LE and TE: 0.05, 0; other parameters were the same as previously described

TribeSim program allows to simulate genetic and cultural evolution of LE and TE (teaching efficiency). Evolution of high‐fidelity teaching is thought by some scholars to be essential for human evolution (Laland, 2017; Morgan et al., 2015). We found that LE can evolve both genetically and culturally (by propagation of the LE memes) with similar success. Interestingly, TE “prefers” to evolve culturally rather than genetically. This is because TE memes have viral properties: When an individual learns a TE meme, she becomes a more efficient machine for meme dissemination. In other words, TE memes are the only memes that help themselves to propagate. When TE is allowed to evolve culturally, TE memes tend to occupy disproportionally large portions of memory (Figure 9). We tentatively suggest that this may have some relation to the fact that human ability to acquire language appears to be mostly congenital (Pinker, 1994), analogous to the LE gene in our model, while the language itself is learned. By learning words and grammar, people acquire the ability to transfer their knowledge to others, that is, to teach; some components of language thus can be regarded as analogs of the TE memes in our simulation.

### 3.6 Additional factors that affect the dynamics of brain‐culture coevolution

**FIGURE 10:**
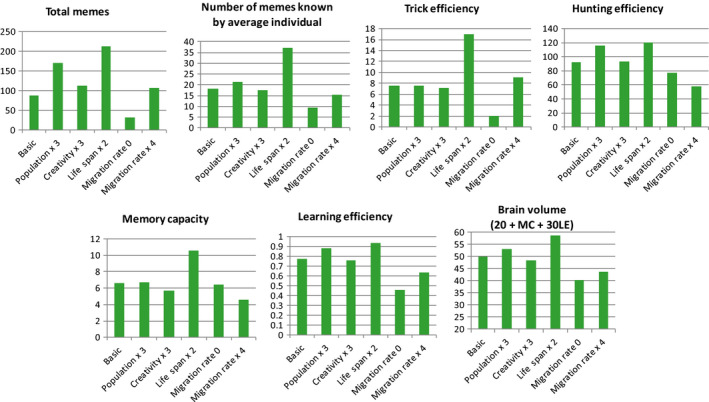
The effects of population size, meme invention rate, life span, and migration rate on brain‐culture coevolution. The results are compared with the “basic” situation (complex culture: TrE, HE, and Useless memes allowed; costly, evolvable LE; G = 40; all other parameters as in Figure 8). Population x 3: the amount of resources available from the environment is multiplied by 3 (R = 9,000; this results in triple population size), other parameters as in the “basic” situation. Creativity × 3: meme invention rate was 0.0004 (instead of 0.000133) per each meme category per year; other parameters as in the “basic” situation. Life span × 2: probability of death per year is Age x 0.0005 (instead of Age x 0.002); this results in average life span 52 years instead of 27 years in the “basic” situation. Migration rate 0: no migration between groups. Migration rate x 4: migration chance was 0.004 per individual per year instead of 0.001 in the “basic” situation. Averages for years 50,000–70,000 from 10 model runs are shown

Finally, we tested the effects of four additional factors: population size, meme invention rate, life span, and between‐group migration (Figure 10).

Larger population size enhances brain‐culture coevolution: The values of brain volume, learning efficiency, hunting efficiency, cultural richness are higher after 50,000–70,000 years of evolution (Figure 10). The effect of higher creativity (higher rate of meme invention, or “asocial learning”) is smaller or even reversed (brain volume, learning efficiency, and memory capacity are slightly lower than in the “basic” situation). This is apparently because highly efficient asocial learning results in a somewhat relaxed need for costly social learning (Muthukrishna et al., 2018). Most importantly, this means that the positive effect of larger population is not explained by higher number of potential inventors (asocial learners), but rather by higher number of brains that can store and disseminate knowledge (and thus by a greater number of opportunities for social learning and lower probability of completely losing a meme) (Henrich, 2015; Kline & Boyd, 2010). This is in concordance with the empirical data showing positive relationships between‐group size and brain size in primates (Dunbar, 2009; Pérez‐Barbería, Shultz, & Dunbar, 2007; Sallet et al., 2011) and between population size and cultural richness in humans (Kline & Boyd, 2010), as well as with the notion that social learning generally may be a much more efficient and evolutionary important strategy than asocial learning (Henrich, 2015; Rendell et al., 2010; Richerson & Boyd, 2005).

Increased life span (Figure 10, life span × 2) results in a much more powerful runaway brain‐culture coevolution, apparently because longer‐lived individuals are more efficient “machines for meme accumulation and dissemination.” This effect is not due to population growth. Doubling of life span results in only 20% increase of population size, because the latter is limited primarily by resources (***R***). Lower death rate automatically results in lower birth rate, because it becomes more difficult for the individuals to accumulate sufficient resources for reproduction. The strong positive effect of longer life span stems from the fact that individuals have more time to accumulate knowledge, so that average individual in the population knows 36–37 memes (instead of 16–17 memes in the “basic” situation). Interestingly, in a population of longer‐lived individuals, the culture becomes considerably more “selfish” (increase in average phenotypic TrE is much higher than in HE, as compared to the “basic” situation [Figure 10]). It is obvious that if we allowed life span to evolve, it would increase in a runaway fashion along with the brain volume and culture, because longer‐lived individuals can acquire more adaptive knowledge and, overall, they benefit more from culture (and especially from the individually beneficial TrE memes). The groups also benefit from the presence of older, more knowledgeable individuals (in TribeSim, such individuals tend to be more efficient “hunters” because they know more HE memes). This would provide an additional positive feedback, further promoting the runaway brain‐culture coevolution. This is in concordance with the fact that humans (including hunter–gatherers with limited or no access to modern medicine) tend to have much longer life spans than any other apes and to retain juvenile characteristics (including enhanced learning ability) into adulthood (Skulachev et al., 2017). More generally, the results are in agreement with the idea that human longevity may be related to the experience and knowledge provided by older members of human groups (Greve & Bjorklund, 2009).

Absence of between‐group migration (isolated groups) generally hinders brain‐culture coevolution, especially the development of Machiavellian culture is compromised (average phenotypic TrE remains low). Conversely, higher migration rate is good for Machiavellian culture, but does not support the development of efficient cooperative culture (HE remains low). This emphasizes the advantage of complex culture, which can ensure powerful brain‐culture coevolution at different levels of between‐group migration as well as at varying levels of between‐group competition (see above).

### 3.7 Comparison between TribeSim and the Cultural Brain Hypothesis (CBH model)

The main similarities and differences between the model presented here (TribeSim) and the CBH model (Muthukrishna et al., 2018) are summarized in Table S2. As can be seen from the table, although both models aim to explore the conditions suitable for the autocatalytic brain‐culture coevolution driven by social learning, the similarities between the two models rarely extend beyond the most general principles and assumptions. Most importantly, TribeSim focuses on the details of cultural evolution (e.g., how different types of adaptive and maladaptive knowledge affect selection for larger brains, how different skills and ideas compete for dominance in memory) and on the effects of between‐group competition. The first aspect is absent in the CBH model in which the “adaptive knowledge” has no internal structure: It is just a number that affects survival and reproduction. Between‐group competition in TribeSim can vary from very strong to absent, while in the CBH model it is weak at most, primarily because there is no resource limitation and the groups do not compete with each other for limited resources. The CBH model is focused on other aspects, such as the evolution of larger group size, oblique social learning (i.e., learning not from genetic parents), learning biases (i.e., the ability to select knowledgeable models to learn from), and evolutionary competition between asocial and social learning.

It should be noted that we designed TribeSim and started to explore its parameter space before the paper by Muthukrishna et al. (2018) was published, and we did not change the design after reading it. The two models thus represent two independent attempts to explore the cultural drive hypothesis by computer modeling. TribeSim can also be regarded as an extension to the CBH model. The main conclusion of both papers is that the autocatalytic brain‐culture coevolution is plausible under specific conditions that appear to be consistent with the current knowledge of hominin ecology, behavior, and evolution. The fact that the same conclusion has been reached on the basis of two very different models implies that it is robust to all the differences listed in Table S2.

The comparison of the more specific conclusions derived from the two models implies that the factors affecting the probability and extent of the runaway brain‐culture coevolution appear to be multiple. However, it remains an open question which of them is most essential. For instance, Muthukrishna and co‐authors emphasized the importance of the “learning bias” (the ability to select knowledgeable models to learn from) and the ability of adaptive knowledge to expand the resource base and thus to increase the carrying capacity of the environment. In TribeSim, both options are absent (individuals select models at random, and the carrying capacity is fixed), but the runaway brain‐culture coevolution is nevertheless possible. Conversely, our results imply that intense between‐group competition, diversity of knowledge (“complex culture”), and the evolution of costly and complexity‐insensitive means of social learning (such as the human language) are powerful drivers of the brain‐culture coevolution and brain expansion. The CBH model does not explore these options, but still it efficiently simulates the cultural drive. Apparently, more work is needed to assess the relative importance of different factors that theoretically can affect the autocatalytic brain‐culture coevolution.

## 4 CONCLUDING REMARKS

Overall, the simulations confirmed the plausibility of the “cultural drive” hypothesis. Under suitable conditions, the runaway coevolution of culture, social learning and brain capacity may start in a social species. The necessary conditions include a minimum starting level of asocial and social learning and a socio‐ecological situation that ensures the possibility of sporadic invention of different, beneficial (for the individual or for the group) and cognitively demanding behaviors (memes). This is similar to the conclusions derived from the CBH model which include “smart ancestors” and “rich ecologies” as necessary preconditions for the runaway brain‐culture coevolution (Muthukrishna et al., 2018).

The chances for the runaway brain‐culture coevolution increase when these sporadically invented memes belong to different categories: Some are individually beneficial (e.g., “Machiavellian”) while the others are group‐beneficial (e.g., “cooperative”). In this case, the runaway brain‐culture coevolution can start and proceed under varying levels of between‐group competition and migration.

Hominins, and especially the Early Pleistocene species of the genus *Homo*, probably found themselves in the right situation due to changes in their social and ecological niche. The changes of the social niche were related to the lower within‐group aggression and competition, higher paternal investment in offspring and the proposed trends toward monogamy, cooperative breeding, and social conformity. This probably resulted in new optimal strategies for reaching higher social status and reproductive success (individuals had to rely more on “Machiavellian intelligence” than on physical strength and violence) (Lovejoy, 2009; Raghanti et al., 2018; Stanyon & Bigoni, 2014). The changes of the ecological niche were related to new feeding strategies such as confrontational scavenging and collective hunting (Bunn & Ezzo, 1993; Stanford, 1999), which required high levels of within‐group cooperation (Bickerton & Szathmáry, 2011; Gavrilets, 2015; Henrich, 2015) and cognitively demanding behaviors such as the Oldowan stone tool making which presumably required high‐fidelity social learning (Morgan et al., 2015).

The simulation results make it possible to suggest a tentative explanation for a somewhat counterintuitive pattern observed in anthropogenesis: In the Early Paleolithic, while cultural development was very slow (e.g., Beyene et al., 2013), the brain volume was increasing rapidly; in the Middle to Late Paleolithic cultural development accelerated greatly, while the brain ceased to increase and even somewhat decreased from the Late Paleolithic to recent (Holloway, 2015). In our simulations, brain expansion was efficiently stimulated by a “rough,” primitive culture consisting of a few large (difficult to learn) memes. Later, as the culture becomes more “sophisticated” (i.e., saturated with numerous small but efficient memes), the brain expansion slows down and even reverses. The mechanism underlying this pattern, which we dubbed “the vicious circle of meme simplification,” requires the combination of two types of limitations: for brain expansion (in TribeSim, larger brains result in lower fecundity) and for the amount of knowledge that can be learned in a lifetime. Another requirement is that the available mechanisms of social learning must give a strong selective advantage to smaller memes. Under such conditions, increasing costs of brain expansion result in stronger selection for smaller memes, which, in turn, makes larger brains less beneficial.

However, brain expansion can receive additional boost if such means of social learning evolve that are at the same time (a) costly in terms of brain expansion (rely on many complicated neuronal circuits) and (b) are tolerant to meme complexity, that is, make it possible to learn and teach difficult skills and concepts relatively easily. Such mechanisms of social learning can break or weaken “the vicious circle,” so that the brain expansion will be able to proceed further. Human language is probably just such a kind of a social learning mechanism. We tentatively suggest that the extraordinarily fast brain expansion in the course of human evolution, which later opened unique possibilities for the development of civilization, was probably a kind of evolutionary accident: The cultural drive was just too strong for the brain to evolve in a more balanced way, for example, by structural optimization rather than by disproportionate increase in volume and the number of cortical neurons.

The results also suggest that the runaway brain‐culture coevolution can acquire additional acceleration from two positive feedback loops: via population growth and via life span extension (e.g., if cultural development results in lower mortality rate) (Figure 10). The former type of a positive feedback (based on the ability of adaptive knowledge to increase the carrying capacity of the environment and, consequently, to result in larger groups and populations) was implemented explicitly in the CBH model (Muthukrishna et al., 2018), but not in TribeSim. This implies that the runaway brain‐culture coevolution is plausible even without such feedback.

The results also imply that between‐group competition (a) tends to make culture less diverse, (b) facilitates the propagation of group‐beneficial memes (Henrich, 2015; Richerson et al., 2016), and (c) effectively removes parasitic memes from the meme pool. Cultural group selection (i.e., selective survival and propagation of groups with more efficient cultures) shapes the content of the meme pool, while individual selection is almost unable to do so. Due to the horizontal spread of memes, culture is a group characteristic rather than an individual one.

## CONFLICT OF INTEREST

The authors declare that they have no competing interests.

## AUTHOR CONTRIBUTION

**Alexander V. Markov:** Conceptualization (lead); Formal analysis (equal); Investigation (equal); Methodology (lead); Supervision (lead); Visualization (equal); Writing‐original draft (equal); Writing‐review & editing (equal). **Mikhail A. Markov:** Formal analysis (equal); Investigation (equal); Software (lead); Visualization (equal); Writing‐original draft (equal); Writing‐review & editing (equal). 

## Supporting information

Table S1‐S2

## Data Availability

TribeSim software is available at Open Science Framework (https://osf.io/cptk6/).
