# Supplementary material for: Runaway brain‐culture coevolution as a reason for larger brains: Exploring the “cultural drive” hypothesis by computer modeling
Source: Ecol Evol. 2020 May 20;10(12):6059–77. doi: 10.1002/ece3.6350 (PMC7319167; doi:10.1002/ece3.6350)
Supplement: Supplementary file 1 — Table S1‐S2 [file ECE3-10-6059-s001.docx]

APPENDIX

**Supplementary Table 1.** The starting set of parameters (used in the first simulation, see section 1 of ‘Results and Discussion’)

| **Parameter** | **Default value** | **Comments** |
| --- | --- | --- |
| Environmental resources per year (R) | 3000 | Maximum amount of resources that can be acquired from the environment per year; puts a limit on population size. Under default parameters, R=3000 means that population size is about 400-500 individuals. |
| Maximum group size (G) | 800 | Under default parameters, G = 800 means that all population is a single group. Population size is always less than 800 due to resource limitation. |
| Memory capacity (MC) gene mutation rate | 0.04 | MC gene mutates with probability 0.04 when transferred from parent to child. |
| MC gene mutation effect Mean, StDev | 0, 0.4 | Mutation effect is added to the gene value. If the resulting gene value is out of the permitted range (in this case, if MC becomes negative), mutation is cancelled and attempt is repeated. |
| All other genes mutation rate | 0 | MC is the only trait that can evolve genetically. |
| Basic brain volume | 20 | Minimum possible brain volume. Brain volume of an individual is 20 when MC gene value is 0. |
| Actual brain volume | 20 + MC | Brain becomes larger by one per each unit of MC |
| Meme categories allowed | TrE, Useless | Individuals can invent and transmit memes for Machiavellian tricks (selfish trait) and memes for Useless actions (maladaptive trait) |
| TrE memes efficiency: mean, StDev | 1, 1.5 | TrE meme efficiency is added to the phenotypic trait TrE of the individual. When a TrE meme is invented, its efficiency is set to normally distributed random value and remains constant thereafter. If meme efficiency is negative, calculation is cancelled and attempt is repeated. |
| TrE meme size: C, R | 1, 2 | Meme size is calculated as the sum of meme efficiency multiplied by C and a random number with zero mean and standard deviation R. If meme size is negative, calculation is cancelled and attempt repeated. |
| Useless memes efficiency: mean, StDev | 0.2, 0.2 | Useless meme efficiency is the probability of performing a useless action per year. If there is more than one Useless meme in memory, their efficiencies are summed as probabilities (e.g., p_1_ + p_2_(1-p_1_)). |
| Useless meme size: C, R | 5, 2 | Coefficients are such that the average sizes of memes of all categories are equal to 1. |
| Useless action cost | 1 | 1 resource is taken from individual for each useless action |
| Probability of forgetting a meme | 0.02 | Individual forgets each meme with probability 0.02 per year |
| Hunting cost | 2 | 2 resources are taken from individual for participating in collective hunting |
| Resources consumed per year (life support) | 3 |  |
| Migration chance | 0.001 | Probability of migration of an individual to another group (per year) |
| Probability of death | Age*0.002 | This results in average life span of about 27 years. |
| Initial state: population size, resources per individual | 10, 20 | Simulation starts with 10 individuals each possessing 20 resources |
| Initial state: LE, TE | 1, 0 | By default, the individuals are perfect learners but incapable teachers. One attempt per year of teaching and learning is performed by each individual. If the transferred meme does not exceed free memory capacity of the learner, learning attempts are always successful, whereas teaching attempts have 0.5 success probability ((teacher’s TE) + (1 – teacher’s TE)*(learner’s LE)). |
| Initial state: HE, TrE, MC | 10, 0, 0 | Individuals initially have hunting efficiency 10 (enough to survive and reproduce under default conditions), Machiavellian trick efficiency 0 and memory capacity 0. |
| Creativity | 0.000266 | Memes of each category are invented with probability 0.000133 per individual per year. Two meme categories are allowed under default conditions, so the total creativity is 0.000266. |

**Supplementary Table 2.** Comparison of the two models describing autocatalytic

brain-culture coevolution: TribeSim (presented in the current paper)

and the “Cultural Brain Hypothesis” (CBH) model by Muthukrishna et al., 2018.

| **Assumptions and principles** | **TribeSim** | **CBH model** |
| --- | --- | --- |
| Individual-based; population consists of groups (demes) | Yes | Yes |
| Larger brain allows for the acquisition and storage of more knowledge | Yes | Yes |
| Large brain is costly | Yes (more resources are needed to produce offspring) | Yes (higher death rate) |
| Increasing costs of larger brains can be offset by more adaptive knowledge | Yes (adaptive knowledge helps to acquire resources needed for costly reproduction) | Yes (adaptive knowledge results in lower death rate) |
| Memory capacity is linked to brain volume and thus costly | Yes | Yes |
| Structure of knowledge | Several types of memes with different effects on individual and group performance; each meme is unique; memes vary in size (complexity) and efficiency, thus allowing for competition and selection at the meme level | Generalized ‘adaptive knowledge’ without internal structure; all knowledge is equally beneficial |
| Details of cultural evolution (e.g., competition between memes) can be traced | Yes | No |
| Effects of different types of knowledge on the brain-culture coevolution can be disentangled | Yes | No |
| Fitness effect of knowledge | Some memes are beneficial for an individual, others are beneficial for a group, still others are maladaptive | All knowledge is beneficial for an individual |
| Beneficial effects of knowledge on survival and reproduction can be disentangled | No. Knowledge affects resource acquisition and thus enhances reproduction and, to some extent, survival (dying of hunger is possible but rare within the explored parameter space) | Yes. Two separate parameters specify the extent to which knowledge affects the number of offspring and the probability of survival to the reproduction stage. |
| Types of learning | Asocial (spontaneous invention of memes), social | Asocial, social |
| Asocial learning | Sporadic, rare; its efficiency is indirectly linked to brain volume because large memes invented by individuals with insufficient memory capacity are immediately forgotten. | Common; its efficiency is linked to brain volume |
| Social learning efficiency (probability of social learning, transmission fidelity) | Evolvable; linked to brain volume and thus costly. | Evolvable; not linked to brain volume. May incur costs due to ‘evolutionary competition’ with asocial learning. |
| ‘Evolutionary competition’ between asocial and social learning | Absent. Social learning proclivity does not affect asocial learning and vice versa. | Present. Individuals that excel in social learning engage in less asocial learning. |
| Learning period | Individuals can learn throughout their lives; learning is generally more efficient in younger age due to more free memory capacity | One or two discrete episodes of learning at the beginning of the life cycle |
| Oblique learning (not from parents) | Always possible | Only during the second learning episode |
| Extension of juvenile period during which the most intensive social learning occurs | Evolves as a consequence of larger memory capacity and richer culture (more time is needed to fill the memory with memes) | The evolving ability to engage in oblique learning (and therefore to use social learning during the second learning episode) is used as a proxy of juvenile period extension |
| Learning bias (ability to select more knowledgeable individuals to learn from) | Not possible, individuals learn from randomly selected group mates | Can evolve |
| Teaching can be disentangled from learning | Yes | No |
| Life cycle | Consists of ‘years’; all principal events and actions (resource acquisition and sharing, learning, teaching, migration, etc.) are repeated every year | Consists of five discrete stages: two episodes of learning, migration, selective death, reproduction |
| Life span | Depends on the parameter that specifies the age-related decrease in survival rate | Not modeled; all individuals go through the same five stages of the life cycle. |
| Genetics | Diploid individuals, sexual reproduction, incomplete dominance, gene recombination | Haploid individuals, clonal reproduction, no recombination |
| Reproductive skew (enhanced reproduction of knowledgeable individuals) | Can evolve as a consequence of elaborate ‘Machiavellian’ culture, wherein only those individuals who know many TrE memes can reproduce efficiently | Specified by a parameter |
| Carrying capacity of the environment; maximum group size | Defined by parameters (R, G), fixed. Adaptive knowledge does not enhance carrying capacity, but instead results in stronger competition for resources. | Increases with the amount of adaptive knowledge |
| Resource limitation | Present; imposed by the environment. The amount of resources that can be extracted from the environment is fixed (R). Groups compete for resources. | Implicitly present; imposed by the level of cultural development. Carrying capacity of the environment increases with the accumulation of adaptive knowledge. Groups do not compete for resources. |
| Between-group competition, group selection | Depends on parameters (R, G); may vary from very strong to absent | Depends on a parameter that specifies reproductive skew (lower skew results in prevalence of group selection); cannot be strong because the groups do not compete for resources |
